# Proximity-Dependent Biotinylation Approaches to Explore the Dynamic Compartmentalized Proteome

**DOI:** 10.3389/fmolb.2022.852911

**Published:** 2022-03-04

**Authors:** Ugo Dionne, Anne-Claude Gingras

**Affiliations:** ^1^ Lunenfeld-Tanenbaum Research Institute, Sinai Health, Toronto, ON, Canada; ^2^ Department of Molecular Genetics, University of Toronto, Toronto, ON, Canada

**Keywords:** proximity-dependent biotinylation, BioID, APEX, post-translational modification, cellular organization, phosphorylation, mass spectrometry

## Abstract

In recent years, proximity-dependent biotinylation approaches, including BioID, APEX, and their derivatives, have been widely used to define the compositions of organelles and other structures in cultured cells and model organisms. The associations between specific proteins and given compartments are regulated by several post-translational modifications (PTMs); however, these effects have not been systematically investigated using proximity proteomics. Here, we discuss the progress made in this field and how proximity-dependent biotinylation strategies could elucidate the contributions of PTMs, such as phosphorylation, to the compartmentalization of proteins.

## Introduction

The functions of proteins are closely associated with their subcellular localization and protein interactions (Bauer et al., 2015; Larance and Lamond 2015; Christopher et al., 2021). Cells can dynamically modify the compartmentalization of proteins in response to environmental changes, rapidly regulating their functions (Bauer et al., 2015). This is generally achieved *via* post-translational modifications (PTMs), which have varied impacts on the targeted proteins (Bauer et al., 2015; Barber and Rinehart 2018). Compartmentalization of cellular proteins in membrane-bound organelles (e.g., mitochondria) and membraneless phase-separated condensates (e.g., nuclear RNA granules) is essential to partition biochemical processes (Lundberg and Borner 2019; Su et al., 2021; Zhang J. Z. et al., 2021). Not surprisingly, protein mislocalization has been linked to many diseases (Hung and Link 2011; Schmidt-Arras and Böhmer 2020).

The compartmentalized proteome has historically been investigated via microscopy-based approaches (Lundberg and Borner 2019; Gnann et al., 2021). In imaging proteomics, libraries of fluorescently-tagged proteins have been used to investigate dynamic protein localization, most notably in yeast (Huh et al., 2003; Tkach et al., 2012; Dénervaud et al., 2013; Chong et al., 2015; Dubreuil et al., 2019). Alternatively, antibodies against endogenous proteins can be used for fluorescence microscopy, and the subcellular location of a large fraction of the human proteome has been determined in this manner by the Human Protein Atlas (Thul et al., 2017; Thul and Lindskog 2018). These approaches allow single-cell analyses and the visualization of intercellular heterogeneity (Gnann et al., 2021); however, they require either the genetic expression of a fluorophore fusion, which can affect protein localization (Dubreuil et al., 2019; Weill et al., 2019), or the availability of specific and validated antibodies (Lundberg and Borner 2019).

Alternatives to microscopy techniques to study organellar organization use biochemical fractionation and mass spectrometry (MS) strategies to assess the localization of large fractions of the endogenous proteome with high throughput and sensitivity (Larance and Lamond 2015). Many organelles can be separated *via* centrifugation, biochemical fractionation, or affinity enrichment approaches followed by MS analyses (Larance and Lamond 2015; Lundberg and Borner 2019; Christopher et al., 2021). While early work required purifying an organelle to near homogeneity prior to MS analysis, more recent studies have harnessed quantitative proteomics workflows and protein correlation strategies to simultaneously define localization across most membranous organelles and even protein complex remodeling (Andersen et al., 2003; Dunkley et al., 2004; Yates et al., 2005; Foster et al., 2006; Kristensen et al., 2012; Christoforou et al., 2016; Itzhak et al., 2016, Itzhak et al., 2017; Jean Beltran et al., 2016; Mulvey et al., 2017; Geladaki et al., 2019; Orre et al., 2019; Heusel et al., 2020; Morgenstern et al., 2021). However, many organelles, particularly membraneless structures, are challenging to specifically separate in intact form, limiting the identification of their components by these approaches. Additionally, protein localization can change following cell lysis and biochemical subcellular fractionation, resulting in imperfect organellar proteome mapping (Larance and Lamond 2015). Despite these limitations, these approaches are extremely powerful at identifying global changes in organellar organization, for example following viral infection (Jean Beltran et al., 2016). In addition to direct assessments of organelle composition by fractionation and MS, protein-protein interaction data has long been used to predict protein localization based on the guilt-by-association principle (i.e., if a protein’s interaction partners localize to a specific organelle, it likely does as well). Recently, leveraging large BioPlex protein-protein interaction (Huttlin et al., 2017) and Human Protein Atlas microscopy-based localization datasets (Thul et al., 2017), a multi-scale integrated cell map was created (Qin et al., 2021). However, identifying protein-protein interactions by affinity purification (AP) coupled to MS suffers from some of the same limitations as classical organellar proteomics—namely, the need to maintain interactions while lysing the cells and purifying the proteins of interest. As we will discuss, covalently labeling proteins in living cells, particularly through proximity-dependent biotinylation (PDB), can bypass some of these limitations, and could potentially also be used to study dynamic and localized PTMs.

Over 500 PTMs have been delineated to date (Keenan, Zachman, and Hirschey 2021), and several alter protein localization. Phosphorylation, perhaps the most studied PTM, is an ideal modification for rapid proteome relocalization, given its prevalence (most eukaryotic proteins can be phosphorylated), dynamic time scale (seconds to hours), and enzymatic specificity (Sharma et al., 2014; Humphrey et al., 2015; Ochoa et al., 2020; Leutert et al., 2021). However, the functional consequences of >95% of identified human phosphosites remain uncharacterized (Needham et al., 2019). In addition, most proteomic approaches determine the global distribution of PTMs but not their subcellular localizations (Altelaar et al., 2013; Larance and Lamond 2015; Lundberg and Borner 2019; Liu et al., 2021). Covalent labeling strategies could bridge this gap in knowledge and highlight the contributions of PTMs, including phosphorylation, to the regulation of protein compartmentalization.

### Proximity-Dependent Biotinylation Approaches

PDB coupled to MS provides an alternative approach to biochemical fractionation strategies to study proteome compartmentalization (Roux et al., 2012; Rhee et al., 2013). We will discuss two frequently used strategies, BioID and APEX; note that singlet oxygen generators (SOG) have also been developed to label proximal proteins with biotin following photoactivation (Glasgow et al., 2016; To et al., 2016; Müller et al., 2021). Both BioID and APEX are based on the genetic fusion of a PDB enzyme to a protein of interest (bait), which is then expressed in a relevant cellular model or organism. Addition of the PDB enzyme substrate induces the covalent tagging of proteins in the bait’s vicinity; these tagged proteins are subsequently captured after cell lysis and identified by MS, negating the need to maintain intact structures during lysis and purification. Like SOG, BioID and APEX rely on the biotinylation of target proteins. Biotin has an extremely high affinity toward streptavidin (Kd ∼10–14 nM), enabling cell lysis and affinity capture under harsh conditions and thus the near complete extraction of a cell’s proteome (Samavarchi-Tehrani et al., 2020). These characteristics of PDB represent important advantages for spatial proteomics.

BioID is based on a mutated abortive biotin ligase from *Escherichia coli* (BirA*, R118G) (Roux et al., 2012). This enzyme generates a “cloud” of reactive biotinoyl-AMP, allowing the covalent labeling of lysine residues on proteins within ∼10 nm of the bait (Roux et al., 2012; Kim et al., 2014). The biotin-labeled proximal interactors are then recovered following harsh cellular lysis via streptavidin affinity and identified by MS (Gingras et al., 2019). BioID can be used in cultured cells or animals, and multiple improvements have been made to the original BirA* enzyme, including implementing biotin ligase systems from other organisms (e.g., BioID2 from *Aquifex aeolicus* and BASU from *Bacillus subtilis* (Kim et al., 2016; Ramanathan et al., 2018)). Additionally, molecular evolution has been used to generate two variants, miniTurbo and TurboID, that have higher activity and allow biotin labeling experiments in the time scale of minutes (Branon et al., 2018), while standard BirA* requires a minimum labeling time of several hours (Youn et al., 2018).

Alternatively, PDB can be achieved with peroxidases. Horseradish peroxidase C (HRP) has been used for protein PDB in cells (Loh et al., 2016; Jiang et al., 2012; Li et al., 2014); however, it is not active in all cellular structures, most notably the cytoplasm (Samavarchi-Tehrani et al., 2020). Ascorbate peroxidases originally engineered for electron microscopy (Martell et al., 2012) have been modified to oxidize biotin-phenol to produce highly reactive phenoxyl radicals, which can covalently biotinylate proximal proteins on tyrosine residues. This approach, termed APEX, can be used in live cells and animals to delineate the proteomes of cellular compartments (Rhee et al., 2013; Hung et al., 2014; Reinke et al., 2017a). As with BioID, APEX uses harsh cell lysis conditions, and proteins are recovered *via* streptavidin affinity purification and identified by MS. One important difference is in the labeling chemistry, as APEX requires hydrogen peroxide treatment. It allows experiments with labeling times as short as 1 min (Rhee et al., 2013). APEX has also been improved, resulting in APEX2, an enzyme suitable for both electron microscopy and proteomics (Lam et al., 2015; Lobingier et al., 2017; Paek et al., 2017).

Although PDB approaches bypass many limitations of other spatial proteomic strategies, they also have intrinsic caveats. For instance, validating that fusing a PDB enzyme to a bait protein (N- or C-termini) does not disrupt its normal biological functions/localization is necessary, and thus labor intensive. Optimisation of the level of expression is also generally required (Christopher et al., 2021). The original BioID method additionally requires long labeling periods that are not compatible with the study of rapid dynamic processes. This issue is attenuated with some of the newer and more active enzymes, though the higher intrinsic affinity for biotin of TurboID in particular may induce endogenous protein biotinylation before biotin treatment and necessitate biotin starvation, which can itself generate stress (Madsen et al., 2015). The hydrogen peroxide used in APEX can have important unwanted cellular effects, such as perturbing phosphorylation (Vepa et al., 1999), making it less desirable for studying this PTM.

### Organelle Mapping With Proximity-Dependent Biotinylation: Current State of the Art

Theoretically, a protein’s proximal interactome can be used as a readout of its cellular environment. As compartments and organelles only contain a fraction of the proteome, it is possible to decipher their composition by directing the PDB enzyme to a specific compartment. Multiple groups sought to characterize organelles by fusing known resident proteins (or shorter amino acid sequences directing their localization) with PDB enzymes, including the nuclear lamina, which was examined in the first BioID study (Roux et al., 2012; Xie et al., 2016). The mitochondrial proteome has been elucidated at subcompartment resolution in live cells (by APEX and BioID) (Rhee et al., 2013; Hung et al., 2014; Han et al., 2017; Antonicka et al., 2020) and in *Drosophila* muscle tissues (by APEX) (C.-L. Chen et al., 2015). APEX was also successfully used to map *Caenorhabditis elegans* proteins that localized to the cytoplasm or nucleus across multiple tissues, demonstrating the feasibility of *in vivo* tissue-specific mapping of organelles (Reinke et al., 2017b). Some groups have investigated organelle proteomes by fusing localization markers to PDB enzymes. For example, a system combining AP-MS and BioID was used to characterize the proximal proteomes of multiple organellar markers (Liu et al., 2018a). Alternatively, the biotinylation patterns detected by densitometric analysis of the streptavidin Western blots of APEX-fused baits have been used to infer their subcellular localization *via* “organelle barcodes” (Lee et al., 2016). PDB enzymes can also map organelle contact sites, where many biological processes occur, which are characteristically difficult to study. For instance, APEX has been used to investigate associations between the endoplasmic reticulum (ER) and the plasma membrane (Jing et al., 2015), to study the cytosol-facing membranes of the mitochondria and the ER (V. Hung et al., 2017), and to map the mitochondrial-autophagosome synapse during mitophagy (Heo et al., 2019).

“Split” versions of PDB enzymes, which have enzymatic activity only when brought in close proximity by pairs of interacting or proximal proteins that are fused to the enzyme halves, are particularly relevant for investigating organellar contact sites (Han et al., 2019; Kwak et al., 2020). Many split-PDB systems have been successfully designed, including split-HRP (Martell et al., 2016), split-APEX (Xue et al., 2017; Han et al., 2019), and split-BioID (De Munter et al., 2017; Schopp et al., 2017; Kwak et al., 2020). These methods have also been used to detect extracellular protein-protein interactions between cells (Martell et al., 2016), intracellular homodimers (De Munter et al., 2017; Xue et al., 2017), and protein complexes (Schopp et al., 2017). The highly active enzyme TurboID was also recently split, allowing the investigation of organelle contact sites and SUMO-dependent interactions (Cho et al., 2020; Barroso-Gomila et al., 2021).

PDB also allows the mapping of specific cellular regions by studying proteins associated with precise subcellular locations distinct from classical membrane-bound organelles. Early examples include the nuclear pore complex (Kim et al., 2014), E-cadherin adherens junction (Guo et al., 2014), paxillin focal adhesion (Dong et al., 2016), cilia, centriole, and centrosome-cilium interface (Firat-Karalar et al., 2014; Gupta et al., 2015; Mick et al., 2015). In addition, PDB enzymes can be targeted to specific genomic loci *via* fusion with dead Cas9 to map neighboring chromatin-interacting proteins (Liu et al., 2018b; Gao et al., 2018; Myers et al., 2018). Impressively, PDB was used directly in the mouse brain to investigate postsynaptic proteomes (Uezu et al., 2016). APEX and BioID have also recently been used to map the proteomes of subcompartments that are historically difficult to isolate, such as lipid droplets (Bersuker et al., 2018) and phase-separated RNA nuclear bodies (Youn et al., 2018, 2019; Markmiller et al., 2018). In addition, the polarized regions of cells have been mapped in organoid models by fusing polarity proteins to APEX, illustrating the utility of PDB to cell-limited models (S. Wang et al., 2021). Interestingly, PDB strategies based on HRP and SOGs are also being developed to map the cell surfaceome (Li et al., 2020; Müller et al., 2021).

Data analysis is an important aspect of using PDB strategies for spatial exploration. For instance, using a prey-centric view of the proteome (i.e., analyzing endogenous preys that are co-labeled by a common set of baits) can increase the specificity and robustness of subcellular localization assignment, provided that a sufficiently high number of baits are used. In this case, incorporating computational and statistical methods based on either simple correlations or factorization approaches permits localization scoring, as recently demonstrated for individual organelles (Youn et al., 2018; Antonicka et al., 2020) and in the generation of a BioID map of close to 4,000 proteins in human HEK293 cells, including >20 intracellular compartments (Go et al., 2021). The selection of the analysis method can influence the ability to score only the preferred localization of a given protein versus uncovering potential instances of multiple localization/moonlighting, as recently discussed (Go et al., 2021). A prey-centric strategy is however only possible when multiple baits are profiled that can help discriminate the labeling profiles of the prey proteins on which they report, making it more difficult to systematically apply across conditions. Lastly, while PDB approaches are clearly compatible with multiple conditions, cell types, tissues and organisms, how to design experiments that will properly capture changes in localization versus proteome compositions will require the development of new experimental and computational strategies.

### A Role for Proximity-Dependent Biotinylation Approaches in Post-Translational Modifications Studies

The studies cited above achieved the impressive feat of delineating spatially resolved subproteomes by PDB. However, these analyses have not typically focused on PTMs due to the requirement of PTMs enrichment strategies to improve coverage of the modified proteome, and the inherent need to use sufficient material when the modifications are substoichiometric and/or dynamic. Thus, the contributions of these important cellular regulatory processes to subcompartment proteomes remain largely uncharacterized. The dynamic re-localization of proteins is controlled by PTMs such as phosphorylation (Deribe et al., 2010; Purvis and Lahav 2013). For example, phosphorylation of ERK and YAP/TAZ regulates their nucleocytoplasmic shuttling (Pocaterra et al., 2020; Guo et al., 2020). Ubiquitination of proteins, e.g., K63, can also alter their intracellular localization (Haglund and Dikic 2005). In addition, phase-separated condensate formation can be stimulated or inactivated by PTMs (Snead and Gladfelter 2019; Su et al., 2021; Tulpule et al., 2021; Zhang J. Z. et al., 2021). Therefore, by simplifying the proteome (and improving peptide coverage of the sampled proteome), PDB-based tools have untapped potential for the study of PTMs in spatial proteomics.

### Combining Proximity-Dependent Biotinylation With Post-Translational Modifications Enrichment Approaches

Specific site-localized PTMs and the enzymes that catalyze them can be studied *via* microscopy (e.g., using PTM-specific antibodies or mutants mimicking or preventing the PTM). This has led, for example, to an understanding of the roles of specific phosphorylation sites in the shuttling of transcription factors and activators between the cytoplasm and the nucleus (Y.-J. Guo et al., 2020; Seif et al., 2017). Microscopy can also be performed at larger scales. For example, the spatial organization of the human kinome was recently revealed by microscopy, localizing 85% (456/538) of human kinases to 10 compartments and highlighting their implications in liquid-liquid phase separation (Zhang H. et al., 2021). Comparatively fewer studies have globally analyzed PTM distributions across organelles to reveal PTM-specific localizations and/or functions, though some studies have begun to shed light on this aspect of regulation (Zhou et al., 2010; Hebert et al., 2013; Still et al., 2013; Christoforou et al., 2016; Krahmer et al., 2018; Lundberg and Borner 2019). For example, a recent study that combined sequential cell fractionation (resolving six subcellular fractions) with phosphopeptide enrichment in cells and mice revealed the rapid relocalization of ribosomal subunits to the nucleolus in response to hypertonicity and muscle contraction, and alterations in the phosphorylation of ribosome assembly factors (Martinez-Val et al., 2021). Specific phosphorylated forms of ribosomal subunits (e.g., ribosomal protein S6 (RPS6) triphosphorylated at S235/S236/S240) were also found in different cell fractions than their nonphosphorylated counterparts (Martinez-Val et al., 2021), suggesting that this strategy can reveal correlations between a protein’s localization and its PTMs, potentially paving the way for mechanistic studies. These studies are however challenging: to simultaneously obtain the organellar separation and the depth of proteome coverage requires access to significant instrument time, and for all procedures to be optimized within a laboratory. Furthermore, how precise are the measurements for a given structure depends on how well it can be isolated from others. Moreover, an outstanding challenge remains to functionally demonstrate that the PTMs identified *via* spatial proteomic strategies regulate the targeted protein’s cellular localization.

**FIGURE 1:**
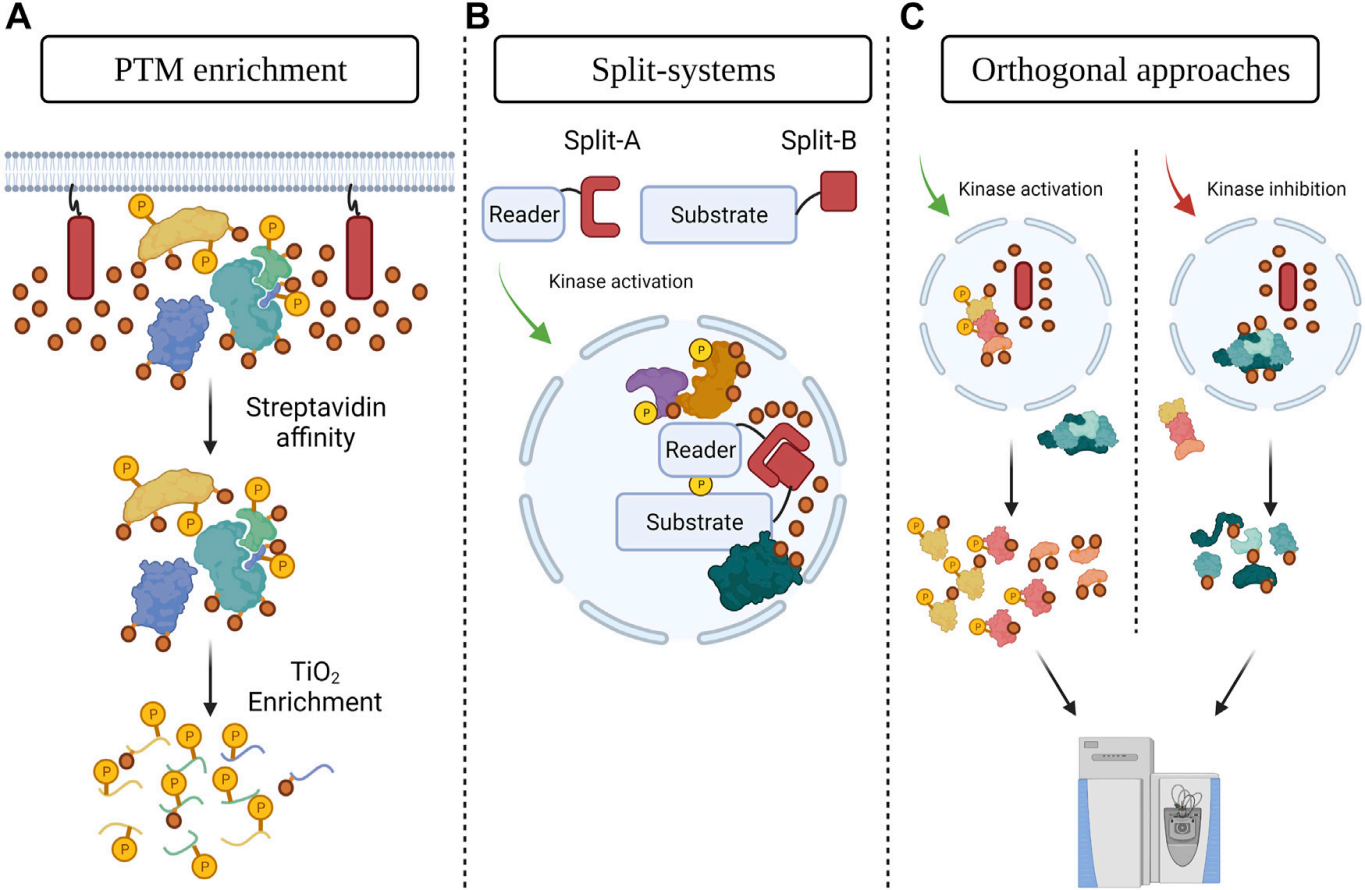
PDB strategies to study the contributions of phosphorylation to proteome compartmentalization **(A)** Combining PDB with phosphopeptide enrichment. A biotinylating enzyme (red rectangle, biotin is represented by orange circles) is targeted to a specific cellular structure to label its spatial subproteome. Streptavidin affinity purification is followed by TiO_2_ enrichment to identify local phosphosites. This concept could be applied to other PTM enrichment strategies as well. **(B)** Protein engineering strategies, such as split-PDB systems, can be used to investigate the consequences of spatially resolved PTMs such as phosphorylation. A reader domain for a specific type of phosphorylation is fused to one half of the PDB enzyme and the substrate of interest (localized to a specific subcompartment) is fused to the other half. The impacts of phosphorylation of the protein of interest can then be determined via PDB-MS. **(C)** Combining PDB tools (red rectangle and orange circles) with orthogonal approaches (curved arrows). PDB is achieved in a specific cellular structure and PTM-generating enzymes are modulated via orthogonal strategies (e.g., optogenetically or chemically). Impacts on the spatial proteome are determined using streptavidin affinity purification and MS. Figure generated with BioRender.com.

Combining PDB with PTM enrichment is a promising means to identify and characterize PTMs with spatial resolution (Figure 1A). For example, organellar or subcompartment labeling *via* PDB strategies combined with phosphoenrichment could identify phosphorylation sites linked to specific subcellular localizations. This can be done either by using the current versions of the PDB systems and enrichment approaches and controlling for their associated backgrounds, or by developing alternative strategies. An interesting recent development was SubMAPP, in which a photoactivatable version of TurboID was localized to the ER, reducing the background signal (Y. Liu et al., 2021). PDB was then performed by combining streptavidin affinity and TiO_2_ phosphoenrichment, which identified almost 1,000 phosphosites in the ER lumen and enabled the authors to monitor the impacts of ER stresses (Liu et al., 2021). Similar strategies could be used to map the phosphorylated proteins of multiple cellular compartments in different conditions, including dynamic membraneless structures.

Split-PDB tools (Figure 1B) would be especially useful to investigate the importance of PTMs for proteome compartmentalization, providing an additional layer of regulatory information. PDB would only occur if the two enzyme halves are brought in close proximity, for example via the interaction of the two fused proteins. The biotinylated proteins would therefore be neighbors of the two interacting molecules and their detection would allow the delineation of complex members in specific cellular compartments. The combination of split-PDB with phosphoenrichment would enable phosphosite mapping at the complex level, hinting at the contributions these modifications make to compartment-specific protein interactions. The split version of the highly active TurboID would be particularly useful to study dynamic PTMs. Again, however, scaling up the experiments to move from a regimen of protein identification/quantification to a specific PTM-bearing peptide identification/quantification will require both optimization and time investment in larger amounts of sample and complex workflows.

### Proximity-Dependent Biotinylation, Orthogonal Approaches, and Protein Engineering to Characterize Compartment-Specific Enzyme Substrates

PDB strategies could also be combined with orthogonal strategies to map the organelle- or subcompartment-specific targets of different enzymes and determine the roles of these PTMs in regulating protein localization (Figure 1C). For example, a strategy termed local kinase inhibition (LoKI) allows the spatial inhibition of protein kinases by localizing inhibitors to subcellular compartments (Bucko et al., 2019). If LoKI was used to inhibit kinases in a specific organelle, the impacts on the local proteome could be characterized by PDB-MS, while organelle-specific substrates of the inhibited kinase would be identified by a PDB and phosphoenrichment workflow. Similar approaches, including targeting chimeras that can dephosphorylate phosphoproteins or degrade kinases and optogenetic tools controlling the spatial inactivation of kinases, could also be combined to PDB-MS to delineate the contributions of specific enzymes or phosphosites to protein subcompartmentalization (Chen et al., 2021; Zhao et al., 2019; Schapira et al., 2019; Huang et al., 2018). Conversely, strategies that allow the spatial activation of enzymes, e.g., light-regulated allosteric switches that control kinase activation optogenetically (Shaaya et al., 2020), may be coupled to PDB. These combinations are not limited to enzymatic PTMs, and PDB could also be coupled to approaches such as T-REX (targetable reactive electrophiles and oxidants) that specifically modifies redox-sensitive proteins via electrophiles (Parvez et al., 2016; Long et al., 2020). While these orthogonal approaches could in principle also be combined with the classical biochemical fractionation strategies described above, their combination with proximity-dependent approaches should enable the study of membraneless organelles and other structures that are difficult to profile through classical approaches.

PTM-regulating enzymes with multi-localization behavior may be targeted to a single location via different protein engineering approaches and fused with a PDB enzyme to identify potential compartment-specific substrates. For instance, many optogenetic tools have been developed to control the specific subcellular localizations of proteins of interest (Toettcher et al., 2013; Zhang and Cui 2015; Buckley et al., 2016; Wang et al., 2016; Benedetti et al., 2018). Chemical inducers can also direct proteins to certain compartments (Stanton et al., 2018). In one study, an inducible association technique was used to localize proteins to the Golgi, the ER, the lysosome, the mitochondria, and the plasma membrane (Komatsu et al., 2010). These tools could be combined with PDB to decipher the proximity interactomes of enzymes regulating PTMs, e.g., kinases and phosphatases, and combined with phosphoenrichment to identify potential organelle-specific substrates.

Another useful approach would be to fuse reader domains (i.e., modules that specifically bind to post-translationally modified proteins) to PDB enzymes and target these fusions to specific compartments. For example, this could allow the mapping of localized phosphorylated proteins using SRC Homology 2 (SH2) or 14-3-3 domains (which recognize pTyr and pSer/Thr, respectively, in specific amino acid contexts). Moreover, applying this concept to engineered broad-spectrum high-affinity reader domains such as the SH2 superbinder, which has been previously used for AP-MS (Kaneko et al., 2012; Bian et al., 2016; Tong et al., 2017), could identify a large fraction of tyrosine-phosphorylated proteins with spatial resolution. An alternative approach would be to engineer variants of biotinylating enzymes that label substrates with specific modifications. This strategy was recently applied to N-terminomics (the study of protein N-termini in the context of mapping the activities of endogenous proteases). A subtiligase (a variant of the serine protease subtilisin) engineered to ligate a peptide ester donor to the N-terminal α-amine of a peptide or protein (Weeks and Wells 2020) was targeted to the plasma membrane. This allowed the ligation of biotinylated peptide esters to the extracellular N-termini of proteins, which were subsequently identified by streptavidin enrichment and MS (Weeks 2021; Weeks et al., 2021).

Split-reporter systems based on protein-fragment complementation assays (PCAs) are extremely powerful tools in molecular biology. Accordingly, many split strategies have been developed to identify binary protein-protein interactions (e.g., yeast two-hybrid (Luck et al., 2020), dihydrofolate reductase (DHFR-PCA) (Tarassov et al., 2008), split-fluorophores (Hu and Kerppola 2003; Paulmurugan and Gambhir 2005; Pratt et al., 2016), and split photosensor domains (Boassa et al., 2019)), to map organelle contact sites (Calì and Brini 2021), and to design cellular signaling biosensors (Linghu et al., 2020; Tenner et al., 2021). Split-PDB systems (described above) could elucidate the roles of PTMs in the formation of subcompartment-specific protein interactions. For instance, orthogonal approaches allowing user-defined kinase or phosphatase activation/inactivation—for example, via optogenetic strategies—could be combined with split-PDB tools to investigate the impact of phosphorylation on spatially resolved protein complexes. In addition, reader domains such as SH2 or the SH2 superbinder (Kaneko et al., 2012; Bian et al., 2016; Tong et al., 2017) could be fused to a split-PDB fragment to delineate the protein interactions associated with specific phosphotyrosine-modified proteoforms (Figure 1C). Like signaling sensors, split-PDB constructs would generate context-dependent signals in specific cellular regions to characterize the impacts of PTMs on the compartmentalized proteome.

## Conclusion

While the study of subcellular proteomes has historically been performed via microscopy and fractionation-MS approaches, PDB has emerged as a powerful strategy to delineate the resident proteins of specific organelles and membraneless condensates. This approach recently led to the determination of a human cell map (https://cell-map.org/) that defined the subcellular localizations of thousands of proteins (Go et al., 2021). While the important impacts of PTMs on protein functions are well known, their contributions to proteome compartmentalization are only beginning to be systematically investigated. PDB has immense potential to bridge this gap and help characterize the roles of PTMs with high spatial resolution, especially for cellular structures that cannot be readily purified from cells. This could be achieved by combining PDB with PTM enrichment methodologies, orthogonal approaches, protein engineering, and split systems. These strategies would be specifically useful to examine dynamic membraneless structures, such as signaling condensates that regulate biochemical processes like actin polymerization and signal transduction (Zhang J. Z. et al., 2021; Su et al., 2021; Case et al., 2019; Huang et al., 2019). Fusing proteins that drive these dynamic structures with biotinylating enzymes to combine PDB with phospho-enrichment could determine the functions of phosphorylation in the assembly and disassembly of phase-separated signaling condensates. It is exciting to imagine all the future applications of PDB in characterizing the roles of PTMs in spatial proteome regulation.
